# Neurofibromatosis type I (NF1) and bone involvement in a pediatric setting: insights from FGF23 levels

**DOI:** 10.1186/s13052-025-01941-9

**Published:** 2025-03-25

**Authors:** Giulia Rodari, Valeria Citterio, Masami Ikehata, Deborah Mattinzoli, Giulietta Scuvera, Federico Grilli, Eriselda Profka, Federico Giacchetti, Valentina Collini, Alessandro Risio, Claudia Cesaretti, Federica Natacci, Carlo Alfieri, Giovanna Mantovani, Claudia Giavoli

**Affiliations:** 1 Endocrinology Unit, Fondazione IRCCS Ca’ Granda Ospedale Maggiore Policlinico, Milan, Italy; 2 Department of Nephrology, Dialysis and Renal Transplantation, Fondazione IRCCS Ca’ Granda Ospedale Maggiore Policlinico, Milan, Italy; 3 Medical Genetics Unit, Fondazione IRCCS Ca’ Granda Ospedale Maggiore Policlinico, Milan, Italy; 4 Department of Clinical Sciences and Community Health, University of Milan, Milan, Italy

**Keywords:** Neurofibromatosis type I, NF1, FGF23, Bone

## Abstract

**Background:**

Neurofibromatosis type I (NF1) is an autosomal dominant disorder characterized by extremely different phenotypes, sometimes including reduced bone mass. The underlying cause of bone impairment in these patients remains poorly understood, especially in children. Previous studies in mice and single reports in NF1 patients with osteomalacia have shown elevated serum FGF23 levels. The aim of this study was to explore for the first time these results in NF1 pediatric patients to eventually provide biological insight into bone involvement in NF1.

**Methods:**

This is an observational, cross-sectional, single-centre study evaluating FGF23/αKlotho levels, as well as other markers of bone metabolism and densitometric parameters in 31 children affected by NF1 and comparing them to 21 age- and sex-matched controls.

**Results:**

We enrolled 31 patients with NF1(M/F 13/18; 11.7 ± 2.9 years). After correction for bone age, BMAD Z-score was < -2SDS in 5/31 patients (16.1%). No difference was found between FGF23 and αKlotho between NF1 patients and controls. No association was found between auxological, biochemical, genetic and radiological parameters and FGF23 values.

**Conclusion:**

In conclusion, this represents the first study assessing FGF23 levels in NF1 children and its possible relationship with decreased bone mineral density. Contrarily to previous observations, no significant differences were found between NF1 patients and controls regarding FGF23 and αKlotho levels. Additionally, there was no clear association between FGF23 and bone involvement, thus suggesting that this phenomenon is not FGF23-driven or FGF23 derangements might occur later in life. Further research is needed to understand the multifactorial mechanisms and determine optimal intervention strategies.

## Background

Neurofibromatosis type I (NF1, OMIM 162200) is an autosomal dominant multisystemic disorder caused by loss of function mutations in the NF1 gene, located at chromosome 17q11.2. NF1 gene encodes for Neurofibromin, a negative regulator of RAS signal transduction pathway involved in cell proliferation, differentiation or survival. This gene is expressed in many tissues, predominantly in the nervous system but also in cartilage and bone [1, 2].

Patients with NF1 can show extremely different phenotypes, ranging from mere skin manifestations (café au lait patches, axillary or groin freckling) or eye involvement (iris Lisch nodules, hamartomas) to cutaneous/subcutaneous neurofibromas, plexiform neurofibromas, optic pathway glioma or malignant peripheral nerve sheath tumors [3]. As far as bone metabolism is concerned, the presentation can range from mild electrolyte and hormonal imbalance (i.e. mild hypophosphatemia or hyperparathyroidism) to skeletal deformities and osteomalacia-like bone lesions, whose etiopathology is still poorly understood [3, 4]. Moreover, the role of loss of function mutations of NF1 gene in osteocytes’ and osteoclasts’ activity is yet to be clarified.

A study conducted on NF1 knocked-out mice evaluated the role of NF1 gene mutations on osteocytes. These mice showed *fibroblast growth factor 23* (FGF23) levels four times higher than controls, accompanied by several alterations of bone metabolism and the subsequent onset of spontaneous fractures [5]. This study indicated that neurofibromin-deficient osteocytes lead to a mineralization defect through the FGF23 signaling pathway and produce spontaneous fractures. Indeed, FGF23 is a bone-derived hormone that regulates phosphorus reabsorption at the renal proximal tubule (by reducing expression of sodium-phosphate cotransporters) and controls vitamin D metabolism (by inhibiting 1-alpha hydroxylase activity and enhancing 24-hydroxylase activity) [6]. Thus, high levels of FGF23 can influence renal phosphate reabsorption, 1,25 (OH)2 vitamin D3 synthesis and therefore gastrointestinal absorption of phosphate, collectively contributing to the development of rickets and osteomalacia.

FGF23 is composed of a hydrophobic region, an amino-terminal one with homology for the FGFs and a carboxyl-terminal region, unique for FGF23, through which the molecule exerts its action interacting with the membrane FGFs receptor (FGFR) [7]. The mature protein (intact FGF23) possesses an arginine-serine domain (179Arg-180Ser) sensitive to proteases, whose proteolytic action cleavages the initial bioactive molecule into an inactive amino-terminal (n-term FGF23) and a carboxyl-terminal (c-term FGF23) fragment. The c-term FGF23 competes with the intact molecule for its binding to FGFR [8]. The intact FGF23 assay binds two epitopes that flank the proteolytic cleavage site between amino acids 179 and 180, thus it primarily detects biologically active, full-length FGF23 [8], otherwise c-term FGF23 assays usually detect both intact and c-term molecules [9].

Inactivating mutations of NF1 gene are known to stimulate RAS and its downstream signaling pathways, such as phosphoinositide 3-kinase (PIK3)/Akt/mTOR and MAPK/ERK [10]. The RAS signaling pathway has already been proposed as a modulator of FGF23, as suggested by the FGF23 increase found in some specific RAS-opaties, such as cutaneous skeletal hypophosphatemia syndrome (CSHS) and keratinocytic epidermal nevus syndrome [11, 12]. Moreover, somatic mutations of the RAS gene in neoplasms can determine paraneoplastic production of FGF23 thus contributing to an osteomalacia-like bone phenotype [13]. Many reports support the role of RAS in reducing FGF23 production throughout the PIK3 (one of the downstream signaling pathways of activated RAS) [14]; however, the specific role of RAS mutations in dysregulation of FGF23 is yet to be confirmed [15, 16].

In this context, a paraneoplastic production of FGF23 by subcutaneous or plexiform neurofibromas has been postulated [17]. Nonetheless, isolated reports showed that FGF23 staining was absent in the neural-derived tumour cells of neurofibroma [18] as well as the resection of a large neurofibroma with increased uptake (as determined by 18FDG PET-CT scan) did not improve hypophosphatemia [19].

Klotho gene family includes three members (α-Klotho, β-Klotho, and γ-Klotho), and represents an additional important factor regulating the action of FGF23 at tissue level. αKlotho is expressed in the kidney and parathyroid glands, where it forms complexes with FGFR1c, FGFR3c, and FGFR4 and serves as the high-affinity receptor for FGF23, thus functioning as an obligate co-receptor for the FGF23 phosphatonin in distal tubules [20].

Membrane-bound αKlotho regulates glycoproteins on the cell surface including ion channels and growth factors. On the contrary, proteolytic cleavage of transmembrane αKlotho by ADAM proteases sheds the soluble αKlotho, which exerts phosphaturic effects rather independently from FGF23 [21]. In animal models, αKlotho has been shown to exert pleiotropic actions, including cytoprotection, anti-oxidation, anti-apoptosis, protection of vessels, promotion of angiogenesis and vascularization, inhibition of fibrogenesis and preservation of stem cells. The exact diagnostic and therapeutic role of αKlotho in humans is not completely elucidated yet [22].

Bone involvement is quite common in patients affected by NF1, with approximately 50% patients displaying skeletal manifestations in childhood such as defects in bone mineralization, chest wall deformity, kyphoscoliosis, and tibial bowing/pseudarthrosis [4]*.*

Studies evaluating bone metabolism in adults affected by NF1 reported decreased bone mineral density and increased fracture risk. On the contrary, data available in children are scanty. A recent meta-analysis evaluating bone status in NF1 children showed that some degree of bone loss could already happen before 18 years old, even though the decrease of BMD may not be of clinical significance [23]. Indeed, a reduced BMD has already been described in NF1 children, with a tendency to worsen with age and pubertal development [24]. Similarly, the few studies assessing fracture risk in children reported discordant results [23].

Though many factors have been studied, such as vitamin D deficiency and hyperparathyroidism, the exact pathophysiological mechanisms for the bone involvement are still unknown and, unlike other NF1 manifestations, the presence of a genotype/phenotype association is yet to be established.

### Purpose of the study

Considering the high levels of FGF23 found in mice knocked-out for NF1 gene and in a NF1 woman with osteomalacia [18], the aim of this study was to investigate these results in NF1 pediatric patients to eventually provide biological insight into the possible pathogenesis of bone involvement in NF1.

## Subjects and methods

This is an observational, cross-sectional, single-centre study. We consecutively enrolled a total of 31 patients with NF-1 (age range 6.6 to 16.6 years), diagnosed according to clinical criteria of the National Institutes of Health (NIH) [25]. Genetic sequence analysis (sequence reference NM_000267.3) was performed on 29 out of 31 patients. The 4 patients who tested negative in the sequencing analysis underwent further molecular characterization through Multiplex Ligation-dependent Probe Amplification (MLPA) and arrayCGH, which revealed a 17q11.2 microdeletion. All patients with other underlying chronic conditions or currently on treatment possibly influencing calcium-phosphate metabolism were excluded (i.e. glucocorticoids, bisphosphonates).

Moreover, 21 age- sex-matched controls referring to the Endocrine Unit for underlying diseases unassociated to bone impairment were enrolled.

Parental written informed consent was obtained from both parents prior to enrolment.

The study was approved by the Ethics Committee of Milan, Area 2, in the session of 8th October 2019 (authorization number 913_2019bis). All procedures performed in this study were in accordance with the ethical standards of the institutional research committee and with the 1964 Helsinki declaration and its later amendments or comparable ethical standards.

### Auxological data

For all study patients standing height, weight, body mass index (BMI) and pubertal development data were collected.

Standing height (HT) was measured with a Harpenden stadiometer. Height, weight and BMI were expressed as standard deviation scores (SDS) according to the World Health Organization (WHO) Growth Charts [26]. Pubertal development was registered following Tanner stages [27].

Mid-parental height (MPH) was calculated as [(mother height + father height) + 12.5]/2 for males and as [(mother height + father height) -12.5]/2 for female patients [28]. MPH distance was defined as the difference between the recorded height SDS and MPH SDS.

Disease-related orthopedic complications and the presence of neurofibromas were also recorded throughout previous orthopedic and dermatologic reports.

### Laboratory data

Biochemical analysis included total calcium, albumin, phosphorus, creatinine, alkaline phosphatase (ALP), 25-hydroxyvitamin D (25OHD3), parathyroid hormone (PTH), intact and c-terminal FGF23 and αKlotho levels.

Total calcium was corrected for serum albumin according to the formula: total calcium + [(4.0–albumin expressed as g/dL) × 0.8]. On urinalysis, measurement of spot urinary calcium, phosphate, and creatinine was performed. Renal tubular maximum reabsorption of phosphate/glomerular filtration rate (Tmp/GFR) ratio was calculated to evaluate renal phosphate transport from the serum and urinary concentrations of phosphate and creatinine. In particular, 25OHD3 levels were measured by commercial radioimmunoassay (Diasorin, Saluggia, Italy), c-terminal FGF23 and intact FGF23 by ELISA (Immutopics, San Clemente, CA, USA), and αKlotho by ELISA (Tecan IBL International GmbH, Hamburg, Germany).

### Instrumental data

Hand and wrist X-ray scan for bone age was assessed in all study patients, according to the standard of Tanner-Whitehouse III [29] or Greulich & Pyle Atlas [30].

Bone mineral density was evaluated through a dual X-ray energy absorption (DXA) of the lumbar spine (L1-L4) and total body less head using a Hologic Discovery machine (software version 3.3 APEX, Delphi W; Hologic Inc.). Bone mass, as measured by DXA, was reported as bone mineral content (BMC, g) or areal BMD (aBMD, g/cm2). Bone mineral apparent density (BMAD, g/cm3) was also determined, thus obtaining a potential correction method in an attempt to approximate the true volumetric density in children with short stature, based on the assumption that the vertebral body is a cube [31]. BMAD was expressed as Z-score according to Crabtree et al. [32]. Low BMD was considered with values of BMAD lumbar Z-scores equal or less than -2 [33]. For all study patients densitometric vertebral fracture assessment (VFA) was performed to rule out the presence of fractures, according to Genant’s method [34].

### Statistical analysis

Continuous variables are shown as mean and standard deviation (SD) in case of normal data or mean and interquartile range (IQR) otherwise. Shapiro–Wilk was used to test if the continuous variables followed a normal distribution. Categorical variables are shown as absolute and relative frequencies. Unpaired t-test (or Mann–Whitney test) was used for comparisons of continuous variables for independent data. To investigate the correlation between two continuous variables, we calculated Pearson’s index (or Spearman’s index). Finally, we implemented univariate linear regression models between radiological parameters and biochemical data. The considered confidence interval was fixed at 95%, and statistical significance was defined for a two-tail *P* < 0.05.

Statistical analysis was performed using Stata 17.0 (StataCorp, College Station, TX, USA).

## Results

We consecutively enrolled 31 patients with NF1 (M/F 13/18; mean age ± standard deviation SD, 11.7 ± 2.9 years, range 6.6–16.6 years) with clinical diagnosis of NF1. Out of 31 patients, 29 underwent genetic sequence analysis (sequence reference NM_000267.3). Of these, 24 presented a likely pathogenic or pathogenic variant according to ACMG criteria and/or the clinical database ClinVar. One patient's variant is currently not reported in the clinical database and is classified as having unknown significance according to ACMG criteria, but it can be considered pathogenic based on transcript analysis, which revealed splicing alteration effects. The 4 patients who tested negative in the sequencing analysis underwent further molecular characterization through Multiplex Ligation-dependent Probe Amplification (MLPA) and arrayCGH, which showed a 17q11.2 microdeletion.

Data about genetic analysis are reported in Table 1.

**Table 1:** Patients’ genetic data *N* = 31

Patient	Mutation	Type	ACMG^a^	ClinVar^b^
1	NM_000267.3:c.663G > A	nonsense	P	P
2	NM_000267.3:c.5681 T > C	missense	LP	P/LP
3	NM_000267.3:c.1885G > A	missense	LP	P
4	NM_000267.3:c.1885G > A	missense	LP	P
5	NM_000267.3:c.2409 + 1G > T	missense	P	P
6	NM_000267.3:c.1885G > A	missense	LP	P
7	NM_000267.3:c.5426G > C	missense	P	P
8	NM_000267.3:c.3525_3526del	frameshift	P	P
9	NM_000267.3:c.3104 T > G	missense	P	P
10	NM_000267.3:c.6365-2A > G	splicing	P	P
11	NM_000267.3:c.3228_3229insACT	In-frame	LP	/
12	NM_000267.3:c.6364 + 4A > G	intronica	LP	P
13	17q11.2 microdeletion	Del		P
14	NM_000267.3:c.5520 T > G	missense	P	LP
15	NM_000267.3:c.3520C > T	nonsense	P	P
16	NM_000267.3:c.1885G > A	missense	LP	P
17	17q11.2 microdeletion	Del		P
18	NM_000267.3:c.4537C > T	nonsense	P	P
19	NM_000267.3:c.5839C > T	nonsense	P	P
20	NM_000267.3:c.1466A > G	missense	P	P
21	17q11.2 microdeletion	Del		P
22	NM_000267.3:c.5248A > G	missense	LP	P/LP
23	NM_000267.3:c.99A > G	sinonima	LP	VUS/P
24	NM_000267.3:c.5944-1G > T	splicing	P	P
25	NM_000267.3:c.2407C > T	nonsense	P	P
26	NM_000267.3:c.4515 G > C; r.4514_4515 ins 14 bp (TTTGCTGTATCTAG) p.Arg1505Ser *53	splicing	VUS	/
27	NM_000267.3:c.1756_1759del	frameshift	P	P
28	NM_000267.3:c.5425C > T	missense	P	P
29	NA			
30	NA			
31	17q11.2 microdeletion	Del		P

^a^*ACMG* American College of Medical Genetics and Genomics guidance for the interpretation of sequence variants

^b^*ClinVar* ClinVar database

Auxological and biochemical data are reported in Table 2.

**Table 2:** Patients’ clinical data (N = 31)

Clinical data	mean ± SD^a^/median(IQR^b^)/ratio
Sex (M/F)	13/18
Chronological age (years)	11.7 ± 2.9
Bone Age (years)	11.1 ± 3.4
Height (cm)	143.1 ± 16.9
Height (SD^a^)	-1.16 (-1.4; 0.21)
BMI^c^ (kg/m^2^)	17.4 ± 2.7
BMI WHO^d^ (SD)	-0.32 ± 0.87
Pre-pubertal/pubertal	8/23

^a^*SD* Standard deviation

^b^*IQR* Inter-Quartile range

^c^*BMI* Body Mass Index

^d^*WHO* World Health Organization

Among them, 8/31 were pre-pubertal (Tanner stage 1) and 2/31 had short stature (HT SDS < -2 SD, 6.4% cases). Neurofibromas were reported in 11/31 patients. None of the children presented vertebral fractures, whereas two patients were affected by scoliosis and one had skeletal abnormalities (ossifying fibroma of the knee).

Among study patients, the majority presented with single-nucleotide mutations, 4/31 reported 17q11.2 deletion.

Data about αKlotho and FGF23 (intact and c-term) values in NF1 patients and controls are provided in Table 3.

**Table 3:** Biochemical data of NF1 patients and controls

Laboratory data	NF1 mean ± SD^a^/median(IQR^b^)	Controls mean ± SD/median(IQR)	P-value
Serum-corrected Calcium (mg/dL, nv 8.5–10.5)	9.27 ± 0.23	9.16 ± 0.43	ns*
Phosphorus (mg/dL)	4.56 ± 0.54	4.61 ± 0.49	ns*
Alkaline phosphatase (IU/L, nv 62–100)	184.6 ± 88.2	198.8 ± 44.1	ns*
Alkaline phosphatase SD^a^	-0.95 (-1.34; -0.44)	-0.76 (-1.43; -0.20)	ns°
25OHvitD^c^ (ng/dL)	30.6 ± 9.9	24.5 ± 8.2	ns*
TmP/GFR^d^	1.63 ± 0.30	1.56 ± 0.32	ns*
FGF23^e^ intact (pg/mL)	41.6 ± 13.9	49.3 ± 12.2	ns*
FGF23^e^ c-terminal (RU^f^/mL)	59.2 (48; 73.1)	62.1 (56.2; 72.2)	ns°
αKlotho (pg/mL)	2132.2 (1678.6; 2973.6)	2578.1 (2182.6; 3228.5)	ns°

^*^T-Student test

°Mann–Whitney test

^a^*SD* Standard Deviation

^b^*IQR* Inter-Quartile range

^c^*25OHvitD* 25-hydroxyvitamin D

^d^*TmP/GFR* Renal tubular maximum reabsorption of phosphate/glomerular filtration rate

^e^*FGF23* Fibroblast Growth Factor 23

^f^*RU* Relative Units

**Fig. 1:**
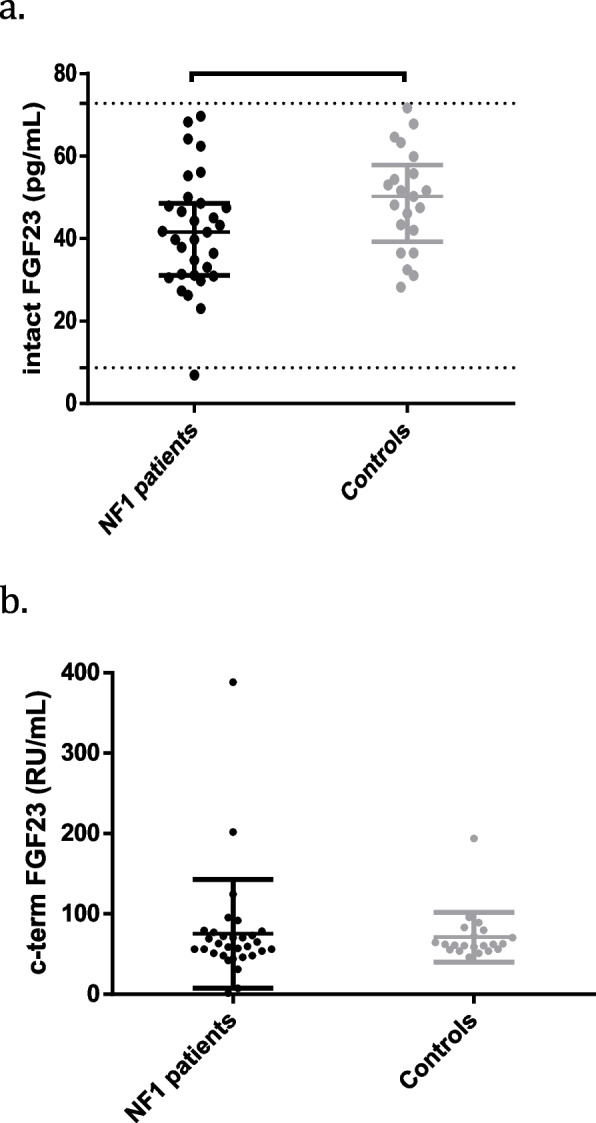
Intact-(**a**) and c-term (**b**) FGF23 levels between NF1 patients and controls

Noticeably, one NF1 patient had intact FGF23 levels < -2 SDS, whereas 3/31 had c-term FGF23 values < -2 SDS and 1/31 over 2 SDS. In controls, 1/31 patients showed c-term FGF23 > 2 SDS. No difference was found between αKlotho, intact FGF23 and c-term FGF23 between NF1 patients and controls (Fig. 1 a, b).

As far as calcium-phosphate metabolism was concerned, 25OHD3 was under 30 ng/mL in 13/31 patients (41.9%) but < 20 ng/mL in 5/31 patients (16.1%), but none under 10 ng/mL, despite supplementation with cholecalciferol in 25/31 (80%) of them.

Hyperparathyroidism was found in 2/31 (6.4%) patients (37.8 and 42.1 ng/L, respectively, nv < 36.8 ng/L), with levels of vitamin D low or within the reference range (26.7 ng/mL and 35 ng/mL, respectively) and normal albumin-corrected calcium levels (9.34 mg/dL and 9.38 mg/dL, respectively). However, both patients had recently started supplementation with cholecalciferol for hypovitaminosis D and in both cases repeated PTH after some months of adequate 25OHD3 levels resulted in the reference range, thus confirming their hyperparathyroidism to be secondary to hypovitaminosis D. In 2/31 cases (6.4%) TmP/GFR was below the reference range, thus indicating renal phosphate wasting. Among them, only one had hypophosphatemia and the other serum phosphorus levels at the lower limit, both with FGF23, αKlotho, 25OHD3 and PTH within the reference range. Among the whole cohort, the only additional patient with hypophosphatemia had normal serum FGF23, αKlotho and PTH levels but hypovitaminosis D (11.8 ng/mL).

No difference was found in TmP/GFR, αKlotho and serum FGF23 levels between patients with hypophosphatemia/phosphorus at lower limit of range and others.

On the other hand, regarding alkaline phosphatase (ALP) levels, 2/31 had ALP SDS < -2 SDS and 2/31 ALP SDS over 2 SDS.

Densitometric data are reported in Table 4.

**Table 4:** Patients’ densitometric data

Data	Mean ± SD
LS^a^ aBMD^b^ (g/cm^2^)	0.62 ± 0.15
LS aBMD Z- score (SD)	-1.14 ± 1.21
LS BMAD^c^ L1-L4 (g/cm^3^)	0.19 ± 0.03
LS BMAD L1-L4 Z-score (SD^d^)	-0.81 ± 1.28
TB aBMD Z-score (SD^d^)	-1.51 ± 1.21

^a^*LS* Lumbar Spine

^b^*aBMD* Areal Bone Mineral Density

^c^*BMAD* Bone Mineral Apparent Density

^d^*SD* Standard Deviation

After correction for bone age, BMAD Z-score was below -2 SDS in 5/31 patients (16.1%) aged between 11.9 and 15.2 years (mean 13.86 ± 1.24 years). None of them reported hypophosphatemia.

**Fig. 2:**
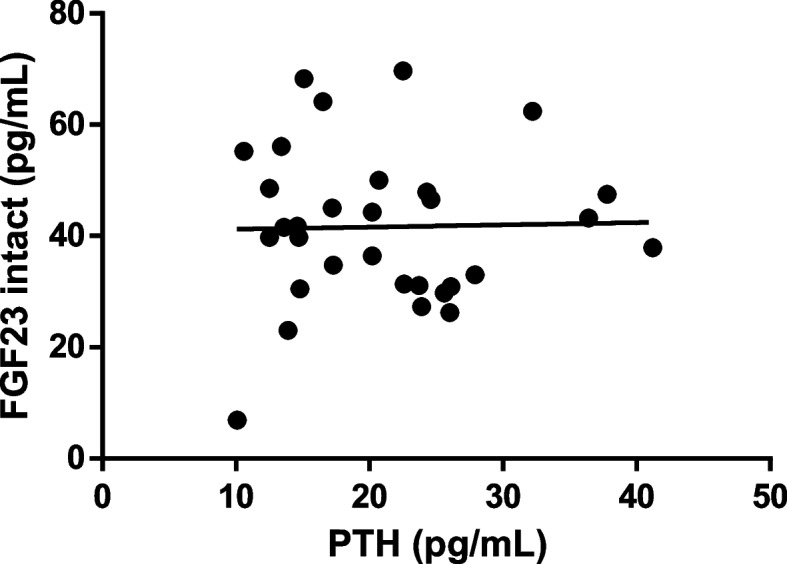
Intact-FGF23 levels at PTH increase

No association was found between auxological, biochemical, genetic and radiological parameters and FGF23 values. Noticeably, PTH and intact and c-term FGF23 concentrations seemed completely independent between each other (Figs. 2**, **and 3).

**Fig. 3:**
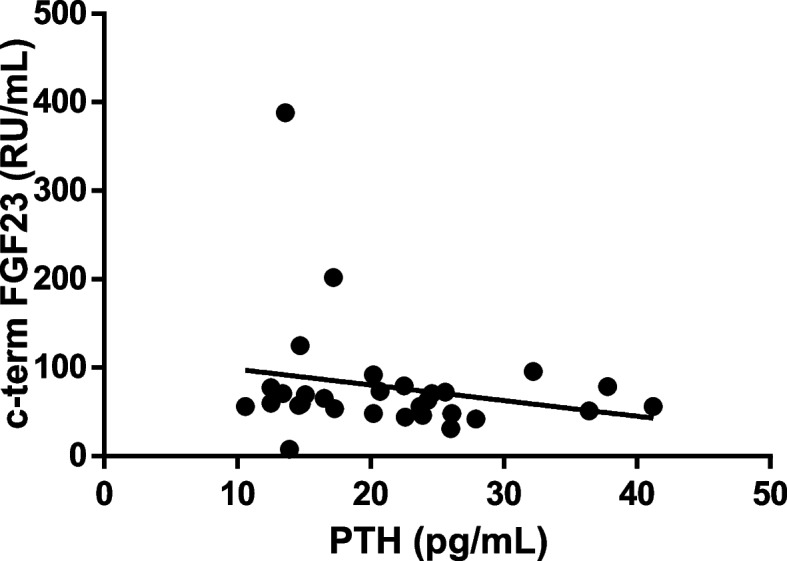
c-term-FGF23 levels at PTH increase

## Discussion

To the best of our knowledge, this represents the first clinical study on FGF23 levels in children affected by NF1.

Preclinical data has have shown that neurofibromin plays an important role in controlling osteocytes FGF23 expression throughout the PI3K pathway. NF1 deficiency in osteocytes produces the overexpression of FGF23 gene and the subsequent significant increase of serum FGF23 levels, thus inducing several mineralization defects, ranging from hyperosteoidosis to a decrease in bone mechanical strength [4].

In contrast to the NF1 deficient mice model of Kamiya et al. [5] and the few clinical reports of specific RAS-opathies (namely CSHS or keratinocytic epidermal nevus syndrome) [11, 12], our study did not find any statistical difference in c-terminal FGF23, intact-FGF23 or αKlotho levels between NF1 patients and controls. Moreover, no association was found between FGF23 values and auxological, biochemical or radiological parameters.

To the best of our knowledge, there are no studies comparing FGF23 levels between NF1 patients and controls. Only one descriptive study assessed FGF23 levels in NF1 affected women reporting no significant difference in FGF23 levels; although it might be stated that it could be considered inappropriately normal in 12 patients who had concomitant hypophosphatemia, thus suggesting a possible underlying calcium-phosphate derangement [35]. Unfortunately, urinary phosphate was not provided.

Some single case reports describe FGF23 levels and hypophosphatemia in patients affected by NF1, hypothesizing a possible paraneoplastic production of FGF23 by subcutaneous or plexiform neurofibromas. Indeed, one report by Sahoo et al. presents a woman with hypophosphatemia and osteomalacia with high levels of FGF23, but the immunostaining for FGF23 in the neurofibroma-cells was absent and the phenotype did not improve after surgery [18]. Another similar report by Obo et al. describes a NF1 female patient with high levels of FGF23 even after the resection of her two largest neurofibromas, which presented only slightly positive results at immunostaining and no clear FGF23 gene expression at RT-PCR [13]. These reports suggest that the increased osteocytes’ production of FGF23 could be the plausible cause of FGF23-induced hypophosphatemia. On the other hand, the possibility that a minor amount of FGF23 is synthesized and secreted from neurofibromas cannot be completely excluded yet.

Noteworthy, all clinical data on FGF23 in specific RAS-opathies (namely CSHS and keratinocytic epidermal nevus syndrome) derive from adult subjects, hence the possibility that the effective dysregulation of FGF23 production could occur only later in life cannot be excluded. In addition, another previous study by our group observed a progressive impairment of bone mineral density with age and pubertal development in patients affected by NF1 [24]. This may support our observation that major bone alterations may not be present during childhood/adolescence, but could progressively develop through time.

Another issue that needs to be addressed is the difference between intact and c-term FGF23 levels. Intact FGF23 represents the biologically active form, with a half-life of approximately one hour. This form is subsequently metabolized into its inactive c-term and n-term FGF23 fragments. The role of FGF23 fragments is not fully understood yet, though c-term FGF23 can compete with the intact molecule for its binding to FGFR, acting as an endogenous inhibitor of αKlotho-FGFR complex formation and subsequent intact FGF23 signaling, which may thus reduce renal phosphate wasting and alleviate hypophosphatemia [36]. Hence, FGF23 cleavage pathways may represent an important natural regulatory mechanism for phosphate control [37]. Nonetheless, as aforementioned, the intact FGF23 assay is more specific, detecting only the circulating active form, otherwise c-term FGF23 assays can detect both intact and FGF23 fragments [38].

To our knowledge, previous studies assessing both the intact and the c-terminal fragment of FGF23 did not report any difference between the two measures. Moreover, the majority of them do not specify which fragment was considered to perform the statistical analysis.

Regarding phosphaturia, our cohort showed TmP/GFR below the reference range in 6.4% cases, thus indicating renal phosphate wasting. Of these two patients, one had hypophosphatemia and the other had serum phosphorus at the lower limit of normal with normal vitamin D values. This tends to support the evidence that vitamin D deficiency does not represent the main cause of hypophosphatemia in NF1 population, as previously reported [39].

Regarding bone metabolism, our data did not show any significant difference in terms of vitamin D in NF1 children compared to controls, although our data are influenced by the cholecalciferol supplementation in the large majority of our patients. Moreover, BMD was not associated with vitamin D values. This is in accordance to a recent metanalysis showing that bone involvement in NF1 patients seems to be due to a dysregulated bone cellular activity independently from vitamin D. Namely, PTH and bone resorption markers (CTX) resulted elevated, whereas vitamin D, calcium, phosphorous, ALP and osteocalcin levels were not significantly altered in the NF1 patients compared with healthy subjects [39]. Nevertheless, in the knocked-out mice model, serum calcium, phosphorus, parathyroid hormone, and vitamin D levels were significantly altered, as a result of the increased bone remodeling due to the higher levels of FGF23 [5]. In this setting the presence of an underlying genotype/phenotype correlation could be hypothesized, though in our study no difference was found between hypophosphatemia, FGF23 levels and NF1 mutation between patients with 17q11.2 deletion and others. Considering the heterogeneity of pathogenic variants in NF1, the sample size of our study was not sufficient to draw definitive conclusions about the possible presence of genotype/phenotype correlations on this topic.

Interestingly, we observed reduced bone mineral density in approximately 16% patients, independently of FGF23 levels. Indeed, a pathological reduction of bone mineral density has already been described in NF1 children, with a tendency to worsen with age and pubertal development [23]. In particular, few other studies performed in NF1 children reported a prevalence of reduced BMD of around 12–14% [24, 40, 41], which is comparable to the one observed in the present one.

A recent review from Charoenngam et al. [23] analyzing those studies assessing bone metabolism in children affected by NF1, described negative lumbar and femoral BMD SDS in affected children, though the confidence interval did not cross the clinical threshold of -2 and therefore the degree of low BMD may not be of clinical significance and should be always interpreted in the light of fracture risk assessment. Anyhow, our observations confirm the less severe BMD involvement in NF1 children compared to adults, in whom the reduced BMD can range from 15 to 57% of patients [42–44].

On the other hand, while multiple studies investigated BMD in patients with NF1, data concerning fracture risk in NF1 children are scanty. Heerva et al. reported that children had a 3.4-fold increased risk of all fractures compared to a control population. Nonetheless, they found only one vertebral fracture in the whole cohort [42]. Other studies did not observe any significant difference in the fracture risk between NF1 children and their siblings or controls, thus supporting our observations, where none of our patients reported any fragility fractures [45, 46].

The main limit of the present study is the small sample size, nonetheless the presented data appeared to be of clinical significance considering the exploratory design, firstly describing FGF23 levels in children with NF1, a rare genetic disease, and insight its possible relationship with decreased bone mineral density.

## Conclusions

Our results tend to exclude a FGF23/αKlotho pathway derangement in NF1 children thus suggesting that the BMD reduction found in a consistent proportion of these patients is not FGF23-driven or that FGF23 impairment can occur later in life.

The underlying multifactorial etiology of impaired bone health in NF1 is not fully understood yet, even in adults, thus more insights into the underlying mechanisms are needed to ascertain the optimal time and type of intervention. For this purpose, it would be interesting to extend the present investigation to a wide cohort of adults affected by NF1.

## Data Availability

All relevant summarized data are within the manuscript. Raw data are available from the corresponding author upon reasonable request.

## Abbreviations

NF1: Neurofibromatosis type I
FGF23: *fibroblast growth factor 23*
PIK3: Phosphoinositide 3-kinase
CSHS: Cutaneous skeletal hypophosphatemia syndrome
HT: Standing height
BMI: Body mass index
SDS: Standard deviation scores
WHO: World Health Organization
MPH: Mid-parental height
ALP: Alkaline phosphatase
25OHD3: 25-Hydroxyvitamin D
PTH: Parathyroid hormone
Tmp/GFR: Phosphate/glomerular filtration rate
DXA: Dual X-ray energy absorption
BMC: Bone mineral content
aBMD: Areal BMD
BMAD: Bone mineral apparent density
VFA: Vertebral fracture assessment
IQR: Interquartile range
